# Novel Divergent Members of the *Kitrinoviricota* Discovered through Metagenomics in the Intestinal Contents of Red-Backed Voles (*Clethrionomys gapperi*)

**DOI:** 10.3390/ijms24010131

**Published:** 2022-12-21

**Authors:** Marta Canuti, Bruce Rodrigues, Andrew S. Lang, Suzanne C. Dufour, Joost T. P. Verhoeven

**Affiliations:** 1 Department of Biology, Memorial University of Newfoundland, 45 Arctic Ave., St. John’s, NL A1C 5S7, Canada; 2 Wildlife Division, Newfoundland and Labrador Department of Fisheries, Forestry, and Agriculture, P.O. Box 2007, Corner Brook, NL A2H 7S1, Canada

**Keywords:** viral metagenomics, virus discovery, deltaflexivirus, tombusvirus, virus taxonomy

## Abstract

Metagenomic methods are powerful tools to investigate viral diversity in biological or environmental samples and to identify previously unknown viruses. We used RNA metagenomics to identify, in the gut of red-backed voles, the nearly complete genomes of two novel members of the *Kitrinoviricota*, a phylum including viruses with positive-sense ssRNA genomes encoding an RNA-directed RNA polymerase. The genome of a novel member of the *Tombusviridae* presented four open reading frames (ORFs); a −1 frameshift is potentially involved in generating the viral replicase. This sequence was part of a phylogenetic clade that did not include any officially classified species. The second genome presented a large ORF coding for a viral polyprotein containing the typical protein domains common to flexiviruses. The sequence clustered with currently known members of the *Deltaflexiviridae*. Both viruses appear to represent the first members of novel species in yet undefined genera. The identified viruses likely originated from the vole diet as members of the two viral families are known to infect plants and fungi, respectively. Investigating public databases demonstrated that a much higher richness than currently recognized exists for these two viral families, highlighting the need to update taxonomy systems and possibly also include genomes identified through metagenomics.

## 1. Introduction

Sequence-independent metagenomic methods are a powerful approach to uncover the microbial diversity within a sample [1]. In particular, this approach has been broadly used to explore the viromes within biological samples collected from a wide variety of organisms, from unicellular populations to vertebrates, and from the environment, allowing the discovery of thousands of previously unknown viruses. Additionally, the diversity of RNA viruses can be more efficiently investigated due to the development of pre-treatment methods that include the degradation of dsDNA, one of the biggest generators of background noise when performing metagenomic sequence analyses [2,3]. The unprecedented increase in our knowledge about viral richness consequent to the introduction of metagenomics was also recognized by the International Committee on Taxonomy of Viruses (ICTV) when the issue of incorporating metagenomic data into viral taxonomy was discussed [4].

The phylum *Kitrinoviricota* (kingdom *Orthornavirae*, realm *Riboviria*) includes positive-sense ssRNA viruses whose genome encodes an RNA-directed RNA polymerase (RdRP) and that infect eukaryotes, including vertebrates, invertebrates, fungi, plants, and protists [5]. The phylum currently includes a total of 4 classes, 6 orders, 21 families, 7 subfamilies, 91 genera, 2 subgenera, and 695 species. However, besides these officially recognized taxa, several other viruses that present genetic similarities to members of this phylum, but that did not yet receive an official taxonomic designation, have been recently discovered [3]. The speed at which novel viruses are discovered indicates that many more currently unknown RNA viruses exist [6] and highlights that more studies will be needed to completely uncover the full spectrum of the diversity of viruses.

In this study, we present the results of an RNA metagenomic investigation performed on a pool of two fecal samples collected from red-backed voles (*Clethrionomys gapperi*) from Labrador, Canada. Among the identified viral hits, the near-complete genomes of two novel viruses belonging to the *Kitrinoviricota* were identified and their genomic features and phylogenetic relationships with other members of their respective families were investigated.

## 2. Results

After a metagenomic investigation performed on RNA isolated from two pooled intestinal samples collected from red-backed voles, we identified 23 contigs (out of the 1935 contigs assembled from the 88,830 obtained reads) showing homology to genomes of viruses from seven different non-phage RNA viral families (Table 1). Most of the obtained contigs were short sequences, representing only a small portion of the viral genome. However, the nearly complete genomes of a novel member of the *Tombusviridae* and of a novel member of the *Deltaflexiviridae* were identified. These two sequences were further molecularly characterized and their phylogenetic placement within the respective viral families assessed.

### 2.1. A Novel Tombusvirus

Among the obtained sequences, we identified a 3879 nt long contig showing homology to members of the *Tombusviridae*, one of the two viral families of the order *Tolivirales*. This family, which includes mainly plant viruses, is divided into three different sub-families, two comprising non-segmented viruses with a genome size between 3.7 and 4.8 kb (*Calvusvirinae* and *Procedovirinae*) and one including viruses with two genomic RNAs of approximately 3.8 kb and 1.4 kb (*Regressovirinae*). These viruses possess a highly conserved polymerase that is encoded by two different ORFs unified either by an in-frame termination codon (amber codon UAG) that can be suppressed to express the full protein or by a −1 frameshifting mechanism [7].

**Figure 1:**
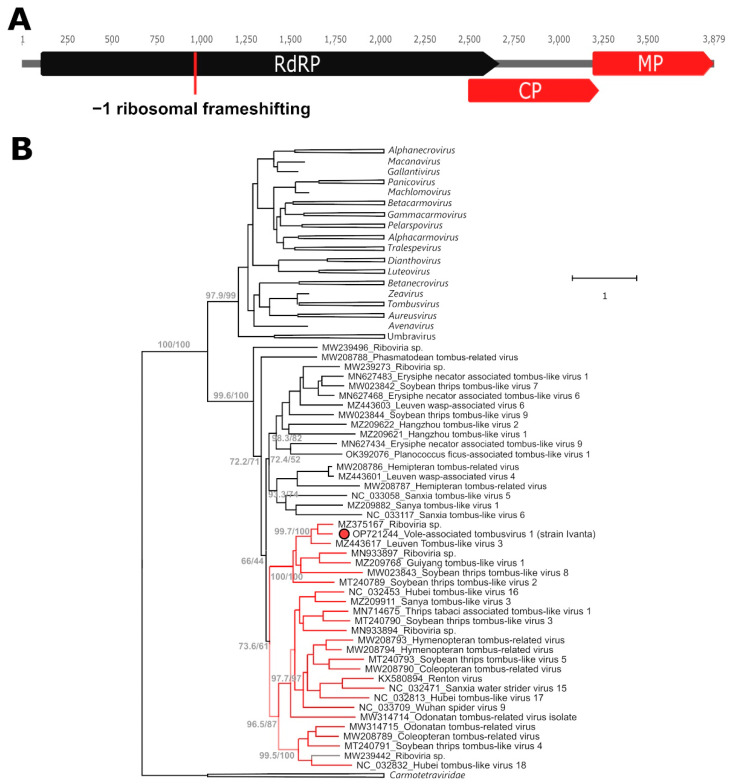
Molecular characterization and phylogenetic analysis of the vole-associated tombusvirus 1 (Va-TV-1). In silico predicted ORFs are shown in panel (**A**) with those coding for the viral replicase (RdRP: RNA-directed RNA polymerase) joined by ribosomal frameshifting shown in black and those encoding the capsid (CP) and movement (MP) proteins in red. The phylogenetic placement of Va-TV-1, indicated by a red dot, within an unclassified clade of the family *Tombusviridae* is illustrated in panel (**B**). The tree is based on the RdRP aa sequence (only the second ORF, alignment of 427 aa) and was built with the maximum-likelihood method based on the LG + F + R10 model with IQ-Tree. The outcomes of the SH-aLRT and bootstrap tests (1000 replicates) are shown for the main nodes. Viruses from the *Carmotetraviridae* and from classified tombusviral genera were used as outgroups and the corresponding clades are collapsed and indicated by triangles. The tree included a total of 78 sequences. Branches of clades with molecular evidence for possible −1 ribosomal frameshifting are labelled in red. The grey branch indicates a short sequence for which the presence of molecular markers for ribosomal frameshifting could not be verified. For each sequence, the accession number and the virus names are indicated.

The vole-associated tombusvirus 1 (Va-TV-1) presented the typical genome organization of several non-segmented members of the *Tombusviridae* and included four ORFs, with the second one encoding the core polymerase containing the canonical “GDD” motif (Figure 1A). Since the stop codon of ORF 1 is TAA and the second ORF has a +2 frame compared to the first ORF, it is possible that this virus uses a −1 frameshifting mechanism to merge the two proteins during translation (predicted final replicase size: 854 aa). A potential heptanucleotide slippery site GAGTTTT, somewhat different from those observed in other tombusviruses [8], was identified 47 nt upstream of the stop codon of ORF 1. Because of sequence homology to proteins of other members of the family, we predict that the remaining two ORFs encode the capsid and movement proteins. Mapping reads to the obtained complete genome showed an average sequencing coverage of 26X and the presence of multiple polymorphic sites, possibly originating from closely related viral variants or from within-host viral mutations.

In a phylogenetic tree built with the partial RdRP of all members of the *Tombusviridae* with the *Carmotetraviridae* as an outgroup (1630 sequences, data not shown), several supported clusters could be noted that did not include any of the officially classified species and corresponded, therefore, to yet-undefined taxa. Va-TV-1 clustered within one of these clades (support: 95.7/99), which also included another 43 yet-unclassified viruses. To further investigate the relationship between Va-TV-1 and these viruses another tree encompassing the almost entire length of the RdRP protein (second ORF) was built with sequences from this clade as well as representative members of all recognized tombusviral genera (Figure 1B). Within the identified clade, we could observe the presence of a subclade of viruses, which also included Va-TV-1, where we found evidence for the potential occurrence of −1 ribosomal frameshift (second ORF in a +2 frame and a NNNTTTT motif at the end of the first ORF), indicating that this mechanism might be evolutionary conserved in this subclade. These sequences are indicated by red branches in the figure. Since for various genera in the *Tombusviridae* the demarcation criterium for species is 75%, based on full replicase and capsid proteins, and since the closest relative to Va-TV-1 is a virus identified in a lizard (*Phrynocephalus erythrurus*) that shares only ~67% identity with Va-TV-1 in these proteins (73.3% identity at the level of the RdRP domain alone, Supplementary Figure S1), Va-TV-1 can be considered a novel species.

### 2.2. A Novel Deltaflexivirus

A 7725 nt long contig showing homology to deltaflexiviruses was obtained. *Deltaflexiviridae* is one of the five viral families within the order *Tymovirales* and it includes fungi-associated viruses (mycoviruses), primarily identified in plant pathogens, plants, and soil. The family, created in 2017, includes only three officially classified species so far, all within one genus (*Deltaflexivirus*), and only a few members have been characterized in more detail [9,10,11].

**Figure 2:**
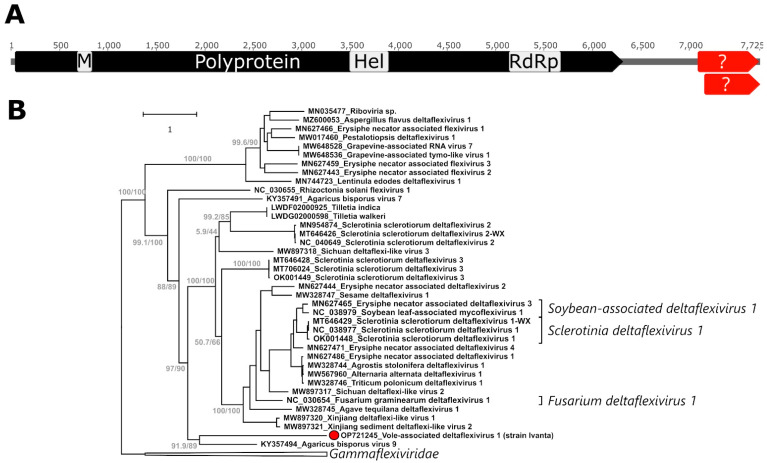
Molecular characterization and phylogenetic analysis of the vole-associated deltaflexivirus 1 (Va-DFV-1). In silico predicted ORFs are shown in panel (**A**) with the one coding for the viral polyprotein in black and identified motifs indicated in grey (M: methyltransferase; Hel: helicase; RdRP: RNA-directed RNA polymerase) and those potentially encoding proteins with no homology to reference sequences in red and indicated by a ?. The phylogenetic placement of Va-DFV-1, indicated by a red dot, within the family *Deltaflexiviridae* is illustrated in panel (**B**). The tree is based on the polyprotein aa sequence (alignment of 3064 aa) and was built with the maximum-likelihood method based on the LG + F + R6 model with IQ-Tree. The outcomes of the SH-aLRT and bootstrap tests (1000 replicates) are shown for the main nodes. Viruses from the *Gammaflexiviridae* were used as an outgroup and the corresponding clade is collapsed and indicated by a triangle. The tree included a total of 42 sequences. For each sequence, the accession number and the virus names are indicated, while species names are indicated on the right, when available.

The vole-associated deltaflexivirus 1 (Va-DFV-1) presented a large ORF, encompassing ~80% of the obtained sequences, coding for the viral polyprotein (2089 aa), which also includes the RdRP (Figure 2A). Although there was a disagreement in the sequence assembly, probably due to the presence of multiple variants in the sample, that prevented from obtaining the terminal 3′ side of the genome, we could identify two additional overlapping ORFs (one partial in 3′) downstream of the main ORF whose predicted protein sequences showed, however, no homology to currently known flexiviral proteins. Comparing this genomic structure with other identified deltaflexiviruses shows that this is a typical genome organization for members of this family, which also possess smaller ORFs in the 3′ region with currently unknown expression potential. The polyprotein contained the typical methyltransferase (aa 214–262), helicase (1150–1282), and RdRP (1699–1875) domains common to all deltaflexiviruses. Mapping reads to the obtained complete genome showed an average sequencing coverage of 93X and the presence of multiple polymorphic sites.

To study the relationship between this virus and other flexiviruses, a phylogenetic tree was built with an alignment of the most conserved part of the RdRP of 3456 sequences of viruses belonging to the order *Tymovirales* (data not shown). In this tree, Va-DF-1 clustered with 38 other sequences (clade support 100/100) in a clade comprising also currently known members of the *Deltaflexiviridae* and two 7.5–8-kb-long contigs obtained from fungal genome scaffolds and labelled in GenBank as belonging to the fungal genome. This clade likely represented the family *Deltaflexiviridae*, and the closest viruses were members of the *Gammaflexiviridae*, with which they formed a supported clade (97.8/100). Based on this analysis, a new tree was built including the majority of the polyprotein sequence, excluding only the beginning section, which was poorly conserved among the different viruses, of the 39 identified deltaflexiviruses and three gammaflexiviruses as an outgroup (Figure 2B). In this tree, the identified virus clustered in a supported clade together with the agaricus bisporus virus 9, its closest relative, whose polyprotein was only ~24% identical to that of Va-DFV-1 (42.7% at the level of the RdRP domain, Supplementary Figure S2). According to the current ICTV criteria for species definition, Va-DFV-1 is therefore the first member of a novel species.

## 3. Discussion

Our knowledge of the diversity of RNA viruses is growing at an unprecedented pace and the number of known species increases every year, with many more viruses still waiting to be discovered [3,6]. The two viruses we characterized in this study belong to two different viral families, *Tombusviridae* and *Deltaflexiviridae*. While the *Tombusviridae* includes several genera and well characterized species [7], the *Deltaflexiviridae* has only been established recently [9] and several aspects regarding the biology and molecular features of its members still have to be elucidated. Both identified viruses, while possessing the key molecular features to be classified in those two families, are divergent from currently known viruses and appear to represent the first members of novel species in yet undefined genera.

Our analyses of sequences available in public databases indicated that the diversity in these two viral families is much higher than has been previously appreciated, with many different possible clusters corresponding to viral genera that have yet to be defined. This highlights how recent advances in metagenomics and sequencing methods have dramatically increased the rate at which viral genomes are discovered. Additionally, as also previously observed [12], existing taxonomy systems should probably be updated to encompass the entire viral diversity, including those viruses for which only the sequence is known. Consistently updating viral taxonomies with novel discoveries is important for our understanding of virus ecology and evolution.

Red-backed voles are rodents that inhabit forests, tundra, and bogs and feed on plants (including leaves, shrubs, berries, roots, nuts), fungi, mosses, lichens, and occasionally insects or slugs [13]. Since the viruses we discovered belong to families that include viruses of plants and fungi [7,9], including plant pathogens, it is likely that Va-DFV-1 and Va-TV-1 are diet-related, although their actual hosts remain unknown. While Va-TV-1 is likely a plant-infecting virus, we suspect that Va-DFV-1 could be a mycovirus infecting a plant pathogen, as many deltaflexiviruses were identified in fungi growing on plants. Interestingly, many of the viruses that were closely related to those found in this study were also identified though metagenomic investigations in biological or environmental samples, and future studies will have to clarify the host and geographic distribution of these viruses. Additionally, further investigations will be required to assess whether members of these two viral families are common in the intestinal content of voles.

Our results further highlight the power of metagenomic investigations in identifying novel viruses but also emphasize their limitations. These methods, in fact, can only identify viral genomes and are not suitable for making assumptions on hosts, pathogenicity, and transmission routes of the viruses they help discover. While sequence and phylogenetic analyses can be precious tools to make hypotheses, and while sequence databases keep growing and a large number of sequences help identify patterns, targeted follow-up experiments are always required to clarify several aspects about the discovered viruses, including their ecology and pathogenic potential [14].

## 4. Materials and Methods

### 4.1. Virus Discovery

This study used samples collected from 2 male red-backed voles (*Clethrionomys gapperi*) trapped in 2017 in Labrador, Canada as part of a provincial small mammal monitoring network. The full intestine was harvested, cut longitudinally, and added to a vial containing 3 mL universal transport medium (Starswab Multitrans System, Starplex Scientific, Etobicoke, ON, Canada). After thorough vortexing, 210 µL of the suspension was pre-treated (centrifugation and DNAse I treatment) and used for nucleic acid (NA) isolation with the DNeasy Blood and Tissue Kit (Qiagen, Hilden, Germany) according to published protocols [15,16]. Subsequently, DNA depletion, retrotranscription, and double-stranded DNA (dsDNA) preparation were performed as described in [17] and dsDNA was outsourced to the Integrated Microbiome Resource of the Centre for Comparative Genomics and Evolutionary Bioinformatics (Dalhousie University, Halifax, Canada) for Illumina high-throughput sequencing after tagmentation-based library preparation (https://imr.bio/protocols.html (accessed on 1 May 2022)).

Trimmomatic (version 0.40) [18] was used in paired-end mode to filter the obtained reads to remove low quality leading and trailing regions (below a quality score of 3), Illumina adapters (using Trimmomatics *IlluminaClip* function), and reads with a length of less than 36 nt. Survived read pairs were assembled into contigs using SPAdes (version 3.15.5, with default settings besides using the *meta-spades* flag) [19,20] and taxonomy was assigned to contigs through BLAST [21] (version 2.13.0+, using the discontiguous megablast option with default parameters except match/mismatch score setting of 1/−1 and a gap existence cost of 2 and extension cost of 1, on the nucleotide collection database downloaded on 24 May 2022). Results were visualized with MEGAN 6 (version 6.19.2) [22] and bacteriophages, including picobirnaviruses [23], were not considered for follow-up analyses.

### 4.2. Sequence Analyses

Read mapping was performed with Bowtie 2 (version 2.3.0) [24] in Geneious R11 (Biomatters), with preset settings “high-sensitivity”, which was also used for genome annotations. Domains were predicted with InterProScan (web-based tool, version 90.0) [25].

A database including all polyprotein sequences of all members of the *Tymovirales*, as annotated in GenBank, and additional viruses related to the discovered deltaflexivirus (identified through BLAST) was built to study the phylogeny of the discovered deltaflexivirus. A database including the RdRP sequences of all members of the *Tombusviridae*, as annotated in GenBank, additional viruses related to the discovered tombusvirus (identified through BLAST), and two sequences from members of the *Carmotetraviridae* to be used as an outgroup was built to study the phylogeny of the discovered tombusvirus. The GenBank database was explored in both cases in September 2022.

Protein alignments were performed with MAFFT (version 7.450, FFT-NS-1 or E-INS-I algorithms for alignments involving more or less than 200 sequences, respectively) [26] and maximum-likelihood phylogenetic trees were built with IQ-TREE 2 (version 2.1.2) [27] with the best model for distance estimates identified with the ModelFinder function [28] as the one with the lowest Bayesian information criterion (BIC). Branch support was assessed using both ultrafast bootstrap approximation (ufBoot) [29] and SH-like approximate likelihood ratio test (SH-aLRT) [30].

## Figures and Tables

**Table 1:** Contigs identified in this study showing homology to RNA viruses.

Contigs		Closest Relative		
Number	Length Range	Virus Name	Family, Genus	Percent Identity
7	257–526 nt	Hubei virga-like virus 11	*Virgaviridae*, unclassified	67–82
6	285–428 nt	various	*Permutotetraviridae*, unclassified	61–69
4	238–1024 nt	various	*Partitiviridae*, unclassified	60–82
3	211–218 nt	Blueberry shock virus	*Bromoviridae*, *Ilarvirus*	80–86
1	243 nt	Israeli acute paralysis virus	*Dicistroviridae*, *Aparavirus*	65
**1**	**3879 nt**	**Riboviria sp. strain 1PE-RDRP-18**	***Tombusviridae*, unclassified**	**67**
**1**	**7725 nt**	**Agaricus bisporus virus 9**	***Deltaflexiviridae*, unclassified**	**69**

Sequences investigated in detail in this study are in bold.

## Data Availability

Sequences obtained in this study have been submitted to GenBank under accession numbers OP721244 and OP721245. The complete genomes were assembled using Illumina reads deposited in GenBank under accession number SRR22012676 (Bioproject: PRJNA893184, Biosample: SAMN31412257).

## Supplementary Materials

The following supporting information can be downloaded at: https://www.mdpi.com/article/10.3390/ijms24010131/s1.
