# Supplementary material for: Novel Divergent Members of the Kitrinoviricota Discovered through Metagenomics in the Intestinal Contents of Red-Backed Voles (Clethrionomys gapperi)
Source: Int J Mol Sci. 2022 Dec 21;24(1):131. doi: 10.3390/ijms24010131 (PMC9820622; doi:10.3390/ijms24010131)

Supplementary Figure S1. Alignment of the RNA-directed RNA polymerase (RdRP) domain of close relatives of the vole-associated tombusvirus 1 (Va-TV-1).

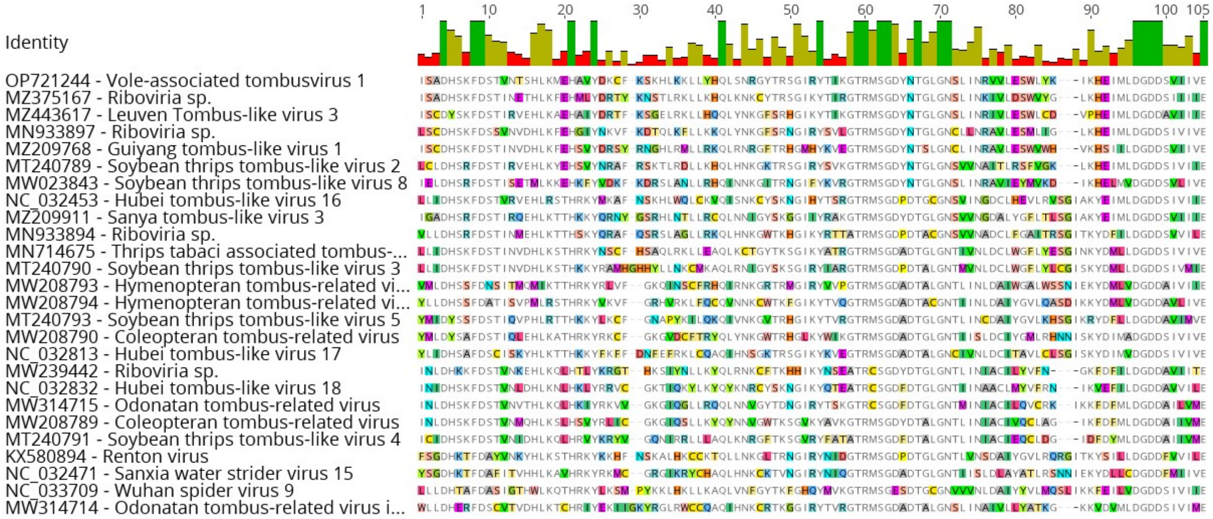

Supplementary Figure S2. Alignment of the RNA-directed RNA polymerase (RdRP) domain of close relatives of the vole-associated deltaflexivirus 1 (Va-DFV-1).

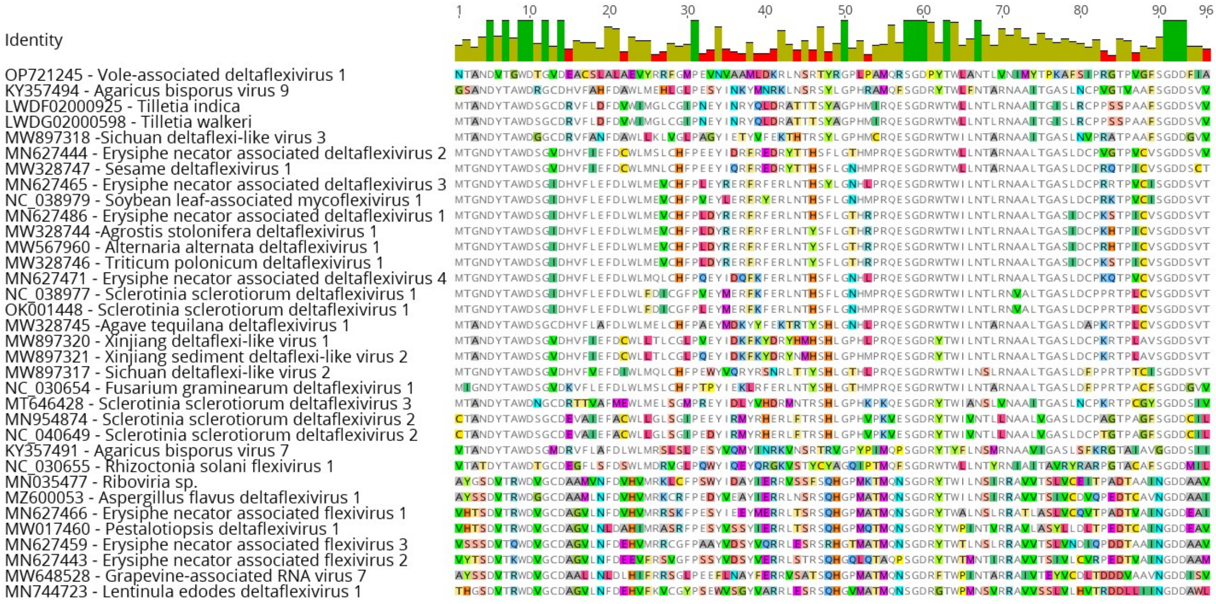

Supplement: Supplementary file 1 [file ijms-24-00131-s001.zip › ijms-2015948-supplementary.pdf]
